# Supplementary material for: Acceptance and compliance with micronutrient powder (MNP) among children aged 6–23 months in northern Nigeria
Source: PLOS Glob Public Health. 2022 Oct 17;2(10):e0000961. doi: 10.1371/journal.pgph.0000961 (PMC10022258; doi:10.1371/journal.pgph.0000961)
Supplement: S1 Dataset — (ZIP) [file pgph.0000961.s008.zip › S8_Dataset/In-depth Interviews.zip!/In-depth Interviews/07072015_A_GA_PM_2_CG.pdf]

INTERVIEW WITH HANNATU NELSON(CAREGIVER)FROM SANSIRI COMMUNITY IN GANYE LOCAL GOVERMENT-ADAMAWA.

Q:DESCRIBE FOR ME THE PEOPLE IN YOUR IMMEDIATE FAMILY LIVING IN YOUR HOUSEHOLD?

R:In this household,it's only me and my husband and my little baby........

Q:HOW OLD IS YOUR BABY?

R:She is 6months going to seven.

Q:WE HAVE HEARED OF FOOD CHALLENGES FOR YOUNG CHILDREN IN THIS SETTING RELATED TO THE SEASONS......WHAT ARE THE COPING MECHANISMS YOU NORMALLY EMPLOY TO ADDRESS THIS CHALLENGES?

R:As a mother,your main responsibility is to take care of the home and maintain the family.personally am a worker(teacher)i earned salary even if it is nothing to write home about.what i normally do during those periods of scarcity is to withdraw money from my savings and assist the family with it.sometimes my husband may lack money,i don't have to wait for him,i get these money then buy the necessary food for my baby and myself,because as nursing mother i need to feed properly and to equally feed and take care of my baby properly.

Q:HOW DOES THISCHALLENGES AFFECT INFANT AND YOUN G CHILD FEEDING?

R:Forme it is not a real problem,because like i told you i normally work....so getting money is not a problem to me....i am only concerned about the rural women who live in the remote villages.This could really be a serious challenge to them because definitely during such hard times they will have no options than to adjust their feeding pattern....may be to either reduce the ration they normally cook or to limit the number of times they do eat in a day....automatically this adjustment will affect the little children food consumption.

Q:ONE THING I HAVE HEARED IN OTHER INTERVIEWS THAT CAREGIVERS OFTEN GIVE THIER CHILDREN 6-23 MONTHS IS TEA....COULD YOU EXPLAIN WHAT THEY MEANT BY "TEA"

R:Well...to my own understading.....tea is very good for a child who has been weaned by the mother,this tea will provide a bit of energy to the child,also tea, is when you mix warm water and bournvita and milk and then feed the child.....but excluding lipton because it is not too good for a little child.bourvita is the best because it will increase the energy and strength of the child.

Q:ASSUMING YOU WANT TO PREPARE "TEA" FOR YOUR BABY HOW WOULD YOU GO ABOUT IT?

R:For me i will only give my child cerelac(baby food) and not tea....because at this age she is not matured enough to drink tea.Alternatively, i will even prefer to give her kunu gyadda mixed with soya beans....because it is more nutritious than ordinary tea.It is not as if i will not give her tea but i will do that less often.

Q:
